# MYD88^L265P^ Detection in IgM Monoclonal Gammopathies: Methodological Considerations for Routine Implementation

**DOI:** 10.3390/diagnostics11050779

**Published:** 2021-04-26

**Authors:** Martina Ferrante, Daniela Furlan, Silvia Zibellini, Michela Borriero, Chiara Candido, Nora Sahnane, Silvia Uccella, Elisa Genuardi, Beatrice Alessandria, Benedetta Bianchi, Barbara Mora, Daniele Grimaldi, Irene Defrancesco, Cristina Jiménez, Federica Cavallo, Dario Ferrero, Irene Dogliotti, Michele Merli, Marzia Varettoni, Simone Ferrero, Daniela Drandi

**Affiliations:** 1 Department of Molecular Biotechnology and Health Sciences, Hematology Division, University of Torino, 10100 Torino, Italy; martina.ferrante@unito.it (M.F.); michela.borriero@unito.it (M.B.); elisa.genuardi@unito.it (E.G.); beatrice.alessandria@unito.it (B.A.); daniele.grimaldi@unito.it (D.G.); f.cavallo@unito.it (F.C.); dario.ferrero@unito.it (D.F.); daniela.drandi@unito.it (D.D.); 2 Department of Medicine and Surgery, University of Insubria, 21100 Varese, Italy; Daniela.Furlan@uninsubria.it (D.F.); silvia.uccella@uninsubria.it (S.U.); 3 Division of Hematology, IRCCS Foundation, Policlinico San Matteo, 27100 Pavia, Italy; S.Zibellini@smatteo.pv.it (S.Z.); kiara-candido@hotmail.it (C.C.); defrancesco.irene@gmail.com (I.D.); M.Varettoni@smatteo.pv.it (M.V.); 4 University Hospital “Ospedale di Circolo e Fondazione Macchi”-ASST Sette Laghi, University of Insubria, 21100 Varese, Italy; nora.sahnane@asst-settelaghi.it (N.S.); benedetta.bianchi90@gmail.com (B.B.); b.mora1988@gmail.com (B.M.); michele.merli@asst-settelaghi.it (M.M.); 5 Hematology Department, University Hospital of Salamanca, Research Biomedical Institute of Salamanca (IBSAL), CIBERONC and Center for Cancer Research-IBMCC (USAL-CSIC), 37001 Salamanca, Spain; jscris@usal.es; 6 Hematology, A.O.U. Città della Salute e della Scienza, University of Torino, 10100 Torino, Italy; 7 Stem Cell Transplant Unit, University Hospital AOU Città della Salute e della Scienza, 10100 Torino, Italy; irenedogl@hotmail.com

**Keywords:** ddPCR, ASqPCR, MYD88, WM, IgM-MGUS

## Abstract

In IgM monoclonal gammopathies MYD88^L265P^ is a prognostic and predictive biomarker of therapy response. MYD88^L265P^ detection is mainly performed by allele-specific quantitative PCR (ASqPCR), however recently, droplet digital PCR (ddPCR) has been proved to be suitable for MYD88^L265P^ screening and minimal residual disease monitoring (MRD). This study compared ASqPCR and ddPCR to define the most sensitive method for MYD88^L265P^ detection in bone marrow (BM), peripheral blood (PB) sorted or unsorted CD19+ cells, and in plasma cell-free DNA (cfDNA). Overall, the analysis showed a good concordance rate (74%) between the two methods, especially in BM samples, while discordances (26%) were mostly in favor of ddPCR (ddPCR+ vs. ASqPCR-) and were particularly evident in samples with low mutational burden, such as PB and cfDNA. This study highlights ddPCR as a feasible approach for MYD88^L265P^ detection across different specimen types (including cfDNA). Interestingly, its high sensitivity makes CD19+ selection dispensable. On the other hand, our results showed that MYD88^L265P^ detection on PB samples, especially with ASqPCR, is suboptimal for screening and MRD analysis. Finally, significantly different MYD88^L265P^ mutational levels observed between Waldenström Macroglobulinemia and IgM monoclonal gammopathy of undetermined significance patients suggest the need for further studies in order to identify possible correlations between mutational levels and risk of progression to Waldenström.

## 1. Introduction

IgM monoclonal gammopathies consist of a broad spectrum of diseases, ranging from apparently benign to malignant conditions, in which variable levels of serum IgM monoclonal proteins are detected [1,2]. The IgM monoclonal gammopathy of undetermined significance (IgM-MGUS) plays a relevant role as a pre-malignant condition, most notably for Waldenström Macroglobulinemia (WM), with a risk of progression of 10% within 5 years from diagnosis [3,4]. IgM-MGUS is defined by the absence of bone marrow (BM) infiltration by lymphoma cells, the presence of a serum monoclonal Immunoglobulin M (IgM) paraprotein lower than 3 g/dL and the absence of end-organ damage [4]. WM is characterized by the heterogeneous infiltration of monoclonal lymphocytes, lymphoplasmacytic cells, and plasma cells in BM, which are responsible for the production of IgM paraprotein in the serum [5,6].

Based on current criteria, the diagnosis of WM requires histological confirmation of monoclonal lymphoplasmacytic cells infiltration by BM aspirate and trephine biopsy: both flow cytometry (MFC) and immunohistochemistry (IC) are advisable in achieving a correct diagnosis [5,6,7,8].

Important progresses in understanding the biology of WM have been made by whole genome sequencing (WGS), that identified MYD88^L265P^ as a recurrent somatic mutation in patients with WM [9,10]. Gradually, several studies using different techniques, such as Sanger sequencing, polymerase chain reaction (PCR), and allele-specific PCR (ASqPCR), confirmed that MYD88^L265P^ is highly frequent in WM (up to 95% of patients) and IgM-MGUS (at least 50%) (Table S1), whereas it is absent in patients with multiple myeloma (MM) (including the IgM isotype) and rarely found in other indolent lymphoid disorders [11,12,13,14,15,16].

It has been shown that mutation status influences clinical presentation and outcome of WM. Indeed, MYD88 wild type (MYD88^WT^) patients are characterized by an inferior overall survival, as well as by higher levels of circulating B cells, positivity for CD23 surface marker and a spectrum of specific additional mutations [17,18]. Moreover, MYD88^WT^ is associated to high rates of progression to symptomatic WM, transformation to aggressive lymphoma, and inferior responses to Bruton’s tyrosine kinase (BTK) inhibitors [17,18,19,20,21,22].

In spite of its diagnostic, prognostic, and predictive role, recognized by the European Society of Medical Oncology (ESMO) clinical guidelines [23], the detection of MYD88^L265P^ has not been standardized yet. A number of distinct assays and methods are available, characterized by different levels of application and sensitivity (Table S1); however, there is lack of consensus about the optimal specimens and techniques, both in terms of operating procedures, test sensitivity, and results interpretation [24,25,26]. Despite ASqPCR currently being the most widely used method, it suffers from some technical restraints. Due to its variable sensitivity (up to 1 × 10^−3^), ASqPCR obtained superior results in highly infiltrated specimens, as BM aspirates (mainly enriched in tumor content by CD19+ cells selection, not routinely practiced in many diagnostic laboratories) [27], while did not perform concordantly well in unsorted BM or peripheral blood (PB) from WM and especially in IgM-MGUS samples (Table S1). Moreover, its suitability for assessing MRD analysis in low infiltrated samples or cell-free tumor DNA (cfDNA) from plasma or other biological compartments, such as cerebrospinal fluid or pleural effusions, need further investigation [28,29,30].

Recently, droplet digital PCR (ddPCR) emerged as a reliable and sensitive diagnostic tool that might overcome some of the ASqPCR limitations, with a higher sensitivity (up to 5 × 10^−5^) [31,32,33]. Moreover, the emerging concept of “liquid biopsy” has been applied also to WM, as a non-invasive, patients friendly, potential alternative to BM aspiration; in fact, plasma has been shown superior to PB, that is hampered by high false negative rates, in particular in patients previously treated with B-cell depleting agents [33,34]. 

Due to the relevance of MYD88^L265P^ both in daily management of WM patients as well as in prospective clinical trial investigating the efficacy of novel agents, it is crucial to find an agreement among different diagnostic laboratories about the most sensitive, appliable, and standardizable molecular technique for mutation detection. 

Thus, the aim of this study was to directly compare the performance of ASqPCR and ddPCR in different tissues and at distinct time points in order to identify the most suitable technique and the most useful specimen for MYD88^L265P^ detection, in order to establish recommendations on laboratory practice for WM and IgM-MGUS marker screening at diagnosis and MRD analysis.

## 2. Materials and Methods

### 2.1. Patients and Samples Collection 

BM and PB samples were collected at baseline and during follow-up (FU) from 227 patients affected by WM and IgM-MGUS. Three patient series, from hematological Italian centers routinely involved in management of IgM monoclonal gammopathies (Torino, Pavia and Varese), were tested for MYD88^L265P^ mutation by both ASqPCR and ddPCR. All patients provided written informed consent for sample collection and analysis (Table S2).

Overall, 319 samples were analyzed including 53 MNC (mononuclear cells), 149 WBC (white blood cells), and 117 CD19+ selected cells (CD19+). Torino series included 62 patients: 60 WM, and 2 IgM-MGUS (105 WBC: 50 BM, 55 PB, (46 at diagnosis, 59 at FU)). Additionally, 64 cfDNA samples extracted from plasma were analyzed. 

Pavia series included 67 patients. Samples from 46 patients were collected at diagnosis, 14 WM, and 32 IgM-MGUS (38 MNC: 31 BM, 7 PB, and 17 CD19+: 11 BM, 6 PB), while a small series of 21 WM patients’ samples were collected before and post-treatment (42 samples: 27 BM-CD19+ and 15 BM-MNC). Varese series included 98 patients at baseline, 64 WM, 34 IgM-MGUS (117 samples: 73 CD19+ (52 BM, 21 PB) and 44 WBC (23 BM, 21 PB)). 

A group of 60 samples, including 40 healthy subjects and 20 MM patients were used as negative control.

For plasma recovery, PB samples, processed within 4 h from the drawing, were centrifuged at 1300× *g* for 13 min at room temperature. Plasma was transferred in separate tubes and centrifuged at 1800× *g* for 10 min at room temperature, before being stored at−80 °C in 1ml aliquots.

BM (previously filtered through a 1ml syringe), PB, and PB leftover (after plasma separation) were treated with erythrocytes lysis buffer (NH4Cl) (at 1:5, 1:2, and 1:2 dilution, respectively), left 15 min at room temperature (lying flat at dark), and centrifuged at 450× *g* for 10 min, at room temperature. The supernatant was discarded and the cell pellet was resuspended in 10–15 mL NH4Cl and centrifuged at 450× *g* for 10 min, at room temperature. The supernatant was discarded and the cell pellet was resuspended in 0.9% NaCl (q.s.), cells were counted, dispensed in 5–10 × 10^6^ stocks, and stored as dry pellets at −80 °C, for further DNA extraction.

MNC B-cells were collected from BM and PB by lymphoprep standard density gradient centrifugation, while CD19+ cells were further isolated from MNCs by immunomagnetic adsorption on MiniMACS separation columns using an anti-CD19 antibody (Miltenyi Biotec GmbH), in accordance with the manufacturer recommendations. The purity of CD19+ separated cells was assessed by flow cytometry using anti-CD19 monoclonal antibodies (Becton Dickinson). 

### 2.2. Nucleic Acid Extraction

Genomic DNA (gDNA) was extracted from WBC, MNC, and CD19+ cells by MaxWell RSC system with blood RSC kit (Promega) (Torino samples), by Puregene Blood DNA isolation kit (Qiagen) (Pavia samples), by Maxwell^®^ 16 LEV Blood DNA Kit (Promega, Madison, WI, USA) (Varese samples), in accordance with the manufacturer recommendations. cfDNA was extracted by Maxwell RSC with LV ccfDNA kit (Promega), in accordance with the manufacturer instructions.

### 2.3. ASqPCR Assays for MYD88^L265P^ Detection

ASqPCR was performed by two assays, according to Jiménez et al., 2014 (Torino) [24] and Varettoni et al., 2013 [16] (Pavia and Varese). Two reverse primers were used by Torino: 5′-CCTTGTACTTGATGGGGATCA-3′ (wild-type-specific reverse primer) and 5′-CCTTGTACTTGATGGGGATGG-3′ (mutant-specific reverse primer) in combination with a common forward primer 5’-ACTTAGATGGGGGATGGCTG-3′ and a specific TaqMan probe (5′-TTGAAGACTGGGCTTGTCCCACC-3′). Pavia and Varese groups performed ASqPCR by SYBR green approach, with two different forward primers 5′-GTGCCCATCAGAAGCGCCT (wild-type-specific forward primer) and 5′-GTGCCCATCAGAAGCGCCC-3’ (mutant-specific forward primer) and a common reverse primer 5′-AGGAGGCAGGGCAGAAGTA-3′.

### 2.4. ddPCR Assays for MYD88^L265P^ Detection

ddPCR mutation detection assay was performed, as previously described by Drandi et al., 2018 [33], using the QX200 Droplet Digital PCR System (Bio-Rad Laboratories, Hercules, CA, USA). Briefly, a single set of primers was combined with two competitive probes, in one assay for MYD88^L265P^ (FAM labeled) and one for MYD88^WT^ (HEX labeled) (CSTM DDPCR HEX/FAM ASSAY BIO-RAD). The cut-off for mutation was settled based on the highest MYD88^L265P^ level detected within the control group. Each experiment included a known highly mutated positive control sample (MUT/WT ratio 6.8 × 10^−1^), a negative control (healthy donor or MM gDNA), and a no template control (NTC). Gate setting was performed based on the positive control results.

All relevant experimental details are reported following the updated digital MIQE2020 (Table S3) [35].

### 2.5. Statistical Analysis 

For methods comparison, ddPCR results were expressed as MUT/WT ratio, while ASqPCR as allele frequency/100. To calculate the correlation and agreement between the methods, we evaluated the test-retest reliability for continuous variables by a single-measurement, consistent, 2-way mixed-effects model, Inter Class Correlation (ICC) analysis, with a 95% confident interval (CI) [36]. Correlation analyses and their representation plots were performed using IBM SPSS Statistics (version 25.0. Armonk, NY, USA: IBM Corp.) or GraphPad5 Software (GraphPad Software Inc., San Diego, CA, USA).

## 3. Results

Overall, 319 samples (239 WM, 80 IgM-MGUS) from 227 patients (159 WM and 68 IgM-MGUS) were tested, in order to identify: (1) the most suitable technique, for MYD88^L265P^ mutation detection between ASqPCR and ddPCR; (2) the most informative specimen for MYD88^L265P^ screening and disease monitoring. To this purpose, different WM and IgM-MGUS tissue samples, such as CD19+ BM sorted cells, WBC, and MNC from BM and PB, were compared by measuring mutation level by both methods. Additionally, a group of 60 samples, including 40 healthy subjects and 20 MM were used as negative controls for both ASqPCR (median ΔCT = 10 (range ΔCT = 8.4–10.7)), setting the cut-off for negativity to ΔCT = 8 and ddPCR (median MUT/WT ratio: 1.75 × 10^−4^ (range 3.10 × 10^−4^–2.70 × 10^−5^)), setting the cut-off for negativity to 3.4 × 10^−4^ [24,33].

**Figure 1:**
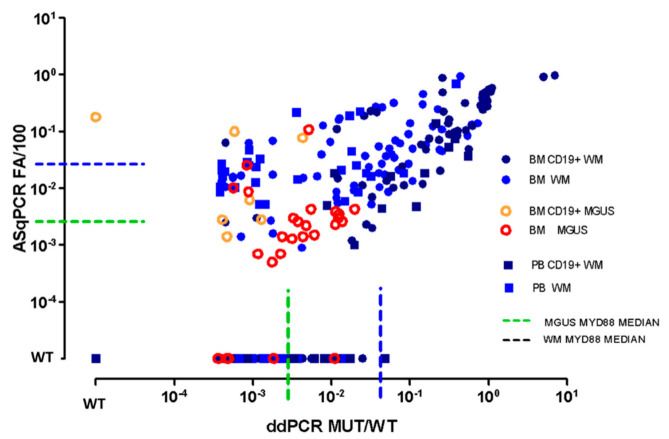
ASqPCR vs. ddPCR. Comparison between ASqPCR and ddPCR for MYD88^L265P^ detection in terms of ratio between MUT and WT. BM: bone marrow; PB: peripheral blood; MGUS: IgM-monoclonal gammopathy of undetermined significance; WM: Waldenström; WT: Wildtype (quantitative value outside the limit of blank. Limit of blank was calculated based on healthy subjects as the mean value +1 SD); 10^−4^: 1 × 10^−4^.

**Figure 2:**
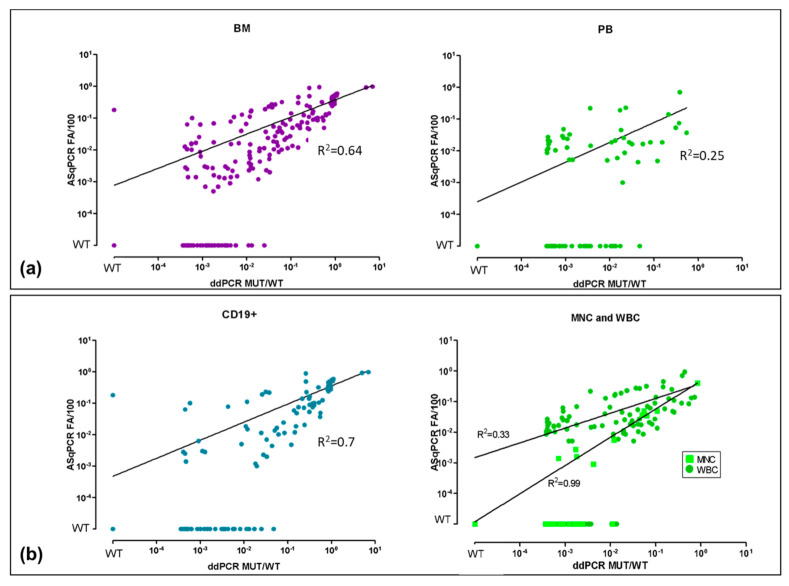
ASqPCR vs. ddPCR in different specimens. Comparison between ASqPCR and ddPCR for MYD88^L265P^ detection in terms of ratio between MUT and WT in (**a**) BM vs. PB and (**b**) CD19+ vs. MNC and WBC. BM: bone marrow; PB: peripheral blood; WT: Wildtype (quantitative value outside the limit of blank. Limit of blank was calculated based on healthy subjects as the mean value +1 SD); MNC: mononuclear cells; WBC: white blood cells; 10^−4^: 1 × 10^−4^.

MYD88^L265P^ was quantified by both ddPCR and ASqPCR on 117 CD19+ selected cells (90 BM, 27 PB), 53 MNC (46 BM, 7 PB), 149 WBC (73 BM, 76 PB). Among them, results from 237/319 samples (74%) were concordant between the two methods: 182 positively concordant and 55 negatively concordant (Figure 1). Among the 182 ddPCR+/ASqPCR+ samples, 141 were BM (106 WM bluish circles, 35 IgM-MGUS reddish circles) and 41 PB (bluish squares: 38 WM, 3 IgM-MGUS), showing a major concordance rate between BM (77%) rather than PB samples (22%). Concordances between methods for each subtypes of samples (BM vs. PB, CD19+ vs. unsorted) are reported in Figure 2, Figures S1 and S2.

Interestingly, no statistically significant differences were detected between WBC (149) and MNC (53) samples, both in terms of concordance between methods (Figure 2b) and tissues (Figure S2). Overall, 58% of WBC (86/149) and 51% of MNC (27/53) were ddPCR+/ASqPCR+, while 21% of WBC (32/149) and 19% of MNC (10/53), were ddPCR-/ASqPCR-. Moreover, to further investigate the differences between WBC and MNC mutation levels, we selected 18 patients whose WBC and MNC samples were collected from the same blood sample showing no discordances in mutation detection between WBC and MNC samples and with a comparable median amount of mutation (3.06 × 10^−2^ WBC, 3.89 × 10^−2^ MNC) (Figure S2). Of note, MYD88^L265P^ levels by ddPCR, seemed to show a superior correlation between sample type compared to ASqPCR. Indeed, among WM samples, BM CD19+ and PB CD19+ (dark blue symbols) showed, as expected, higher mutation levels (MUT/WT > 1 × 10^−2^), compared to BM WBC/MNC and PB WBC/MNC (light blue symbols). Specific clusters between BM and PB were not detectable, however, a high proportion of PB WBC/MNC (light blue squares), showing a lower mutation level compared to BM, clustered around 1 × 10^−3^ MUT/WT ratio level, by ddPCR. Finally, IgM-MGUS samples showed a comparable distribution to WM but with 1 log lower mutation levels (Figure 1, dashed green line vs. dashed blue line).

Within the 55 samples scored negative by both techniques, 21 (38%) were BM (14 WM, 7 IgM-MGUS), while 34 (61%) were PB (26 WM, 8 IgM-MGUS), highlighting the lower mutational load of PB.

Concerning the discordant samples, 82/319 (26%), 81 were ddPCR+/ASqPCR- (35 PB (28 WM, 7 IgM-MGUS) and 46 BM (27 WM, 19 IgM-MGUS)), while only one IgM-MGUS sample was ddPCR-/ASqPCR+. Regretfully it was not possible to replicate the analysis to verify whether it was the case of an ASqPCR false positivity or a ddPCR false negativity (Figure 1). Of note, discordances between ddPCR and ASqPCR were mostly related to samples analyzed by SYBR green approach (Table S4).

### 3.1. ddPCR vs. ASqPCR in Different Tissues (BM vs. PB) 

Considering BM and PB separately, we observed 162/209 (78%) concordant BM: 141 were ddPCR+/ASqPCR+ (median 4.1 × 10^−2^ (range: 4.0 × 10^−4^, 7.1 × 10^1^)), 21 ddPCR-/ASqPCR- and 47 (22%) were discordant. Among discordant BM, 46 were ddPCR+/ASqPCR-, while only one (CD19+) was ddPCR-/ASqPCR+ (median 1.2 × 10^−3^ (range: 3.6 × 10^−4^, 2.5 × 10^−2^)) (Figure 2a and Figure S1a). 

Within PB, 75/110 (68%) were concordant: 41 ddPCR+/ASqPCR+ (median 1.1 × 10^−2^ (range: 3.8 × 10^−4^, 5.5 × 10^−1^)) and 34 ddPCR-/ASqPCR-, while 35 (32%) were discordant (all ddPCR+/ASqPCR-) (median 8.6 x 10^-4^ (range: 3.7 × 10^−4^, 4.8 × 10^−2^)) (Figure 2a and Figure S1b). Notably, ddPCR quantification in concordantly positive samples (ddPCR+/ASq CR+) showed a higher mutation load in BM and PB compared to the ddPCR+/ASqPCR- (median by ddPCR: 4.1 × 10^−2^ vs. 1.2 × 10^−3^ for BM and 1.1 × 10^−2^ vs. 8.6 × 10^−4^ for PB).

### 3.2. ddPCR vs. ASqPCR in CD19+ Sorted vs. Unsorted Samples 

Focusing on CD19+ selected samples, 82/117 (70%) were concordant: 69 ddPCR+/ASqPCR+ (median 2.2 × 10^−1^; range: 4.1 × 10^−4^, 7.1 × 10^−1^) and 13 ddPCR-/ASqPCR-; while 35 (30%) were discordant, 34 ddPCR+/ASqPCR- (median 1.5 × 10^−3^; range: 3.6 × 10^−4^, 4.8 × 10^−2^) with only one ddPCR-/ASqPCR+ (Figure 2b and Figure S2a,b). 

Since no difference in terms of mutation level was observed between WBC and MNC (Figure S2c), we considered both samples in the analysis. Among 202 WBC/MNC samples 155 (77%) were concordant 113 ddPCR+/ASqPCR+ and 42 ddPCR-/ASqPCR- while 47 (23%) were discordant and all ddPCR+/ASqPCR-. Of note, MYD88^L265P^ level was lower in WBC/MNC compared to CD19+ selected cells (median 3.65 × 10^−3^ vs. 3.15 × 10^−2^) (Figure 2b and Figure S2a,b). 

### 3.3. ddPCR vs. ASqPCR in WM Compared to IgM-MGUS

As already described in literature, 93/97 (96%) BM WM and 54/62 (87%) BM IgM-MGUS at diagnosis were mutated by ddPCR, while 79/97 (81%) WM and 36/62 (58%) IgM-MGUS by ASqPCR [11,16,33].

Overall, considering BM, PB, diagnostic and FU samples, ddPCR detected MYD88^L265P^ in 83% (199/239) of WM and 80% (64/80) of IgM-MGUS, while ASqPCR in 60% (144/239) and 48% (39/80), respectively.

**Figure 3:**
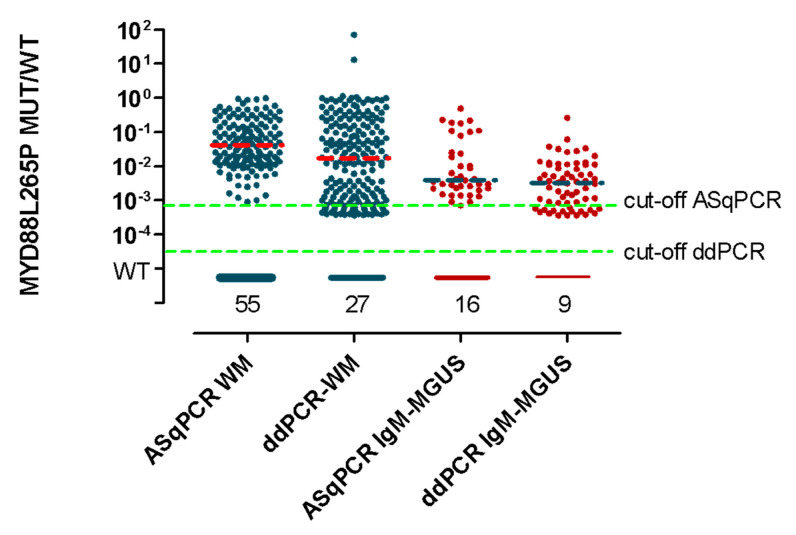
Mutational load comparison between WM and IgM-MGUS. The dashed orange and blue lines show, respectively, the median of MYD88^L265P^ mutational values in WM and IgM-MGUS patients. WT: Wildtype (quantitative value outside the limit of blank. Limit of blank was calculated based on healthy subjects as the mean value +1 SD); 10^−4^: 1 × 10^−4^.

Interestingly IgM-MGUS, showed a lower median mutational level (Figure 1 dashed green line, Figure 3 dashed blue lines) by ddPCR 5.2 × 10^−3^ (range: 3.6 × 10^−4^, 2.6 × 10^−1^) compared to WM (Figure 1 dashed blue line, Figure 3 dashed orange lines) 1.7 × 10^−2^ (range: 3.6 × 10^−4^–7.1 × 10^1^) (*p* < 0.0001). 

### 3.4. ddPCR vs. ASqPCR in Plasma-cfDNA 

**Figure 4:**
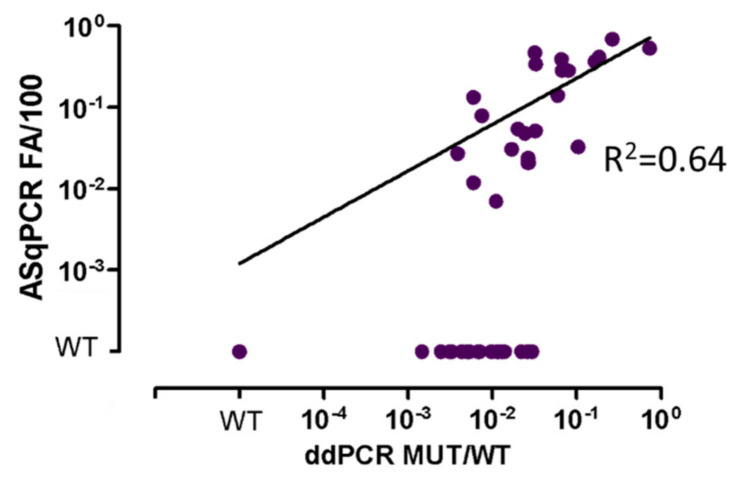
ASqPCR vs. ddPCR in cfDNA from plasma samples. Comparison between ASqPCR and ddPCR for MYD88^L265P^ detection in terms of ratio between MUT and WT in 32 WM, 4 IgM-MGUS, and 28 Lymphomas, 47 at diagnosis and 17 at FU. WT: Wildtype (quantitative value outside the limit of blank. Limit of blank was calculated based on healthy subjects as the mean value +1 SD); 10^−4^: 1 × 10^−4^.

Finally, in order to test the sensitivity of ddPCR on liquid biopsy analysis, we evaluated 64 cfDNA from plasma (47 at diagnosis and 17 at FU) in 32 WM, 4 IgM-MGUS and 28 other B-lymphoproliferative disorders (i.e., central nervous system lymphoma, marginal zone lymphoma, chronic lymphocytic leukemia) (Figure 4 and Figure S3). MYD88^L265P^ was detected in 66% (42/64) of samples by ddPCR (25 WM, 3 IgM-MGUS and 14 other B-lymphoproliferative disorders), while only 36% (23/64) of samples scored positive by ASqPCR, confirming the higher sensitivity of ddPCR compared to ASqPCR even in plasma-cfDNA samples.

## 4. Discussion

In this study ASqPCR and ddPCR were compared in order to define the abilities and aptitudes of each method for MYD88^L265P^ mutation detection and quantification in different tissue samples, such as BM, PB, and plasma-cfDNA, in sorted or unsorted CD19+ cells, collected from WM and IgM-MGUS patients routinely followed in clinical practice in three Italian hematological centers. 

Overall, the analysis showed a good concordance rate (74%) between the two methods, especially in BM samples, while discordances (26%) were mostly in favor of ddPCR (ddPCR+ vs. ASqPCR-). Notably, ddPCR showed a higher sensitivity in detecting MYD88^L265P^ in samples with low mutational burden, such as PB, where median mutational levels were lower than in BM (Figure S1).

Moreover, by selecting CD19+ cells no differences in frequency of patients carrying the MYD88^L265P^ were observed in comparison to WBC or MNC, even if the mutation level was of course different. As expected, both BM and PB CD19+ cells showed a higher mutational burden compared to unselected MNC/WBC samples. Thus, despite enriching the sample in tumor cells content, cell selection is dispensable for mutational screening by ddPCR, due to its superior mutation detection ability in unsorted samples, compared to ASqPCR. 

Concordantly to published literature, in our series ASqPCR detected MYD88^L265P^ in diagnostic BM samples in 81% of WM and 58% of IgM-MGUS, however ddPCR detected the mutation in 96% (93/97) of WM and 87% (54/62) of IgM-MGUS. This makes ddPCR assay ideal for diagnostic use in clinical routine, avoiding the costs and technical requirements for CD19+ cell sorting [11,16,27,33].

The concept of the “high sensitivity” of ddPCR is relevant in the context of treatment tailoring based on patients’ genotype. Recent studies evaluated the efficacy and safety of novel BTK inhibitors (i.e., acalabrutinib, zanubrutinib) on MYD88^WT^ WM patients, so defined by ASqPCR in unsorted BM aspirates, with a declared limit of detection of 0.5% (5 × 10^−3^). Scientists must be aware that the misclassification of patients with a mutation level below the sensitivity of the employed PCR method may lead to an overestimation of the efficacy of novel treatments. As a consequence, the results of the direct comparison between ASqPCR and ddPCR in unselected samples underline how ddPCR could benefit patients stratification for treatment assessment [37,38]. 

This study also highlights a significantly different mutation level between WM and IgM-MGUS. Interestingly, IgM-MGUS showed a median mutation level almost one logarithm lower than WM. This difference is statistically significant and draws attention to the need for further studies on larger patients’ series to identify possible correlations between mutational levels and risk of progression to WM.

Finally, the analysis of 64 plasma-cfDNA confirmed the role of ddPCR for mutational screening and MRD monitoring on less-invasive tissue sources, representing an attractive alternative to BM biopsy particularly in asymptomatic patients, such as those affected by IgM-MGUS. Actually, this study describes the sensitivity of ddPCR for MYD88^L265P^ mutation detection in a group of samples collected in clinical routine. Of note, a multicenter clinical trial for non-invasive diagnostics and monitoring of MRD in WM and in IgM-MGUS patients is currently ongoing, in the context of the Fondazione Italiana Linfomi (BIO-WM trial: NCT03521516), with the primary endpoint of demonstrating that the MYD88^L265P^ mutation rate detected in plasma by ddPCR is superimposable to the rate detected in BM.

## 5. Conclusions

ddPCR is a feasible approach for MYD88^L265P^ detection, more sensitive than ASqPCR across different specimen types (including plasma-cfDNA) and in distinct diseases. MYD88^L265P^ detection on PB samples, especially with ASqPCR, is suboptimal for MYD88^L265P^ screening and MRD analysis. CD19+ selection, despite enriching the sample tumor content, is dispensable for mutational screening, suggesting that the implementation of ddPCR assay in routine diagnostic laboratories might avoid the need of CD19+ selection. There is a need for further studies on larger patients’ series, in order to identify possible correlations between mutational levels and risk of progression to WM. Based on the above conclusions we finally propose an algorithm for the best use of the most convenient PCR methods for MYD88^L265P^ detection, based on the available type of specimens (Table 1) in order to establish a uniform testing approach for determining the MYD88 mutational status.

These recommendations, if followed, will improve routine clinical practice within different laboratories with the intention to standardize protocols and procedures for the management of both clinical routine and multicenter clinical trials.

## Figures and Tables

**Table 1:** Sensitivity and capability of ASqPCR and ddPCR methods for MYD88^L265P^ detection in WM and IgM-MGUS in different specimens. Comparison between ASqPCR and ddPCR for MYD88^L265P^ detection in terms of sensitivity based on the type of sample analyzed and recommendations for using one of the methods based on the type of sample available. Of note, regardless of the sensitivity of the method, PB samples show a lower MYD88^L265P^ mutational load compared to BM. BM: bone marrow; PB: peripheral blood; WBC: white blood cells; CD19+: CD19 selected cells; DIA: diagnosis; FU: Follow-up; cfDNA: cell free DNA.

**WM**	**BM DIA**		**BM FU**	**PB DIA**		**PB FU**	**PLASMA**
	WBC	CD19+	WBC	WBC	CD19+	WBC	cfDNA
ddPCR	**++**	++	++	**++**	++	+	++
ASqPCR	+	**++**	+	+	**++**	+/−	+/−
**IgM-MGUS**	**BM DIA**		**BM FU**	**PB DIA**		**PB FU**	**PLASMA**
	WBC	CD19+	WBC	WBC	CD19+	WBC	cfDNA
ddPCR	**++**	++	++	+	+	+	+
ASqPCR	+/−	**++**	+/−	+/−	+	+/−	+/−

Method sensitivity and detection rate: ++ 5 × 10^−5^, + >1 × 10^−3^, +/− ≤ 1 × 10^−3^, bolded ++ Recommended sample, □ Not recommended method.

## Data Availability

Not applicable.

## Supplementary Materials

The following are available online at https://www.mdpi.com/article/10.3390/diagnostics11050779/s1, Figure S1: ASqPCR vs. ddPCR in BM compared to PB, Figure S2: ASqPCR vs. ddPCR in CD19+ sorted (a) vs. unsorted (b) BM and PB samples and comparison between MNC and WBC (c), Figure 3S: ASqPCR vs. ddPCR in cfDNA from plasma samples, Table S1: Different methods used for MYD88^L265P^ detection in WM and IgM-MGUS in the last 10 years, Table S2: Cohort of patient samples collected for MYD88^L265P^ mutation analysis by ddPCR and ASqPCR, Table S3: digital MIQE2020, Table S4: Discordances between ddPCR and ASqPCR methods within the three series.
