# Supplementary figures and images for: MYD88L265P Detection in IgM Monoclonal Gammopathies: Methodological Considerations for Routine Implementation
Source: Diagnostics (Basel). 2021 Apr 26;11(5):779. doi: 10.3390/diagnostics11050779 (PMC8146978; doi:10.3390/diagnostics11050779)

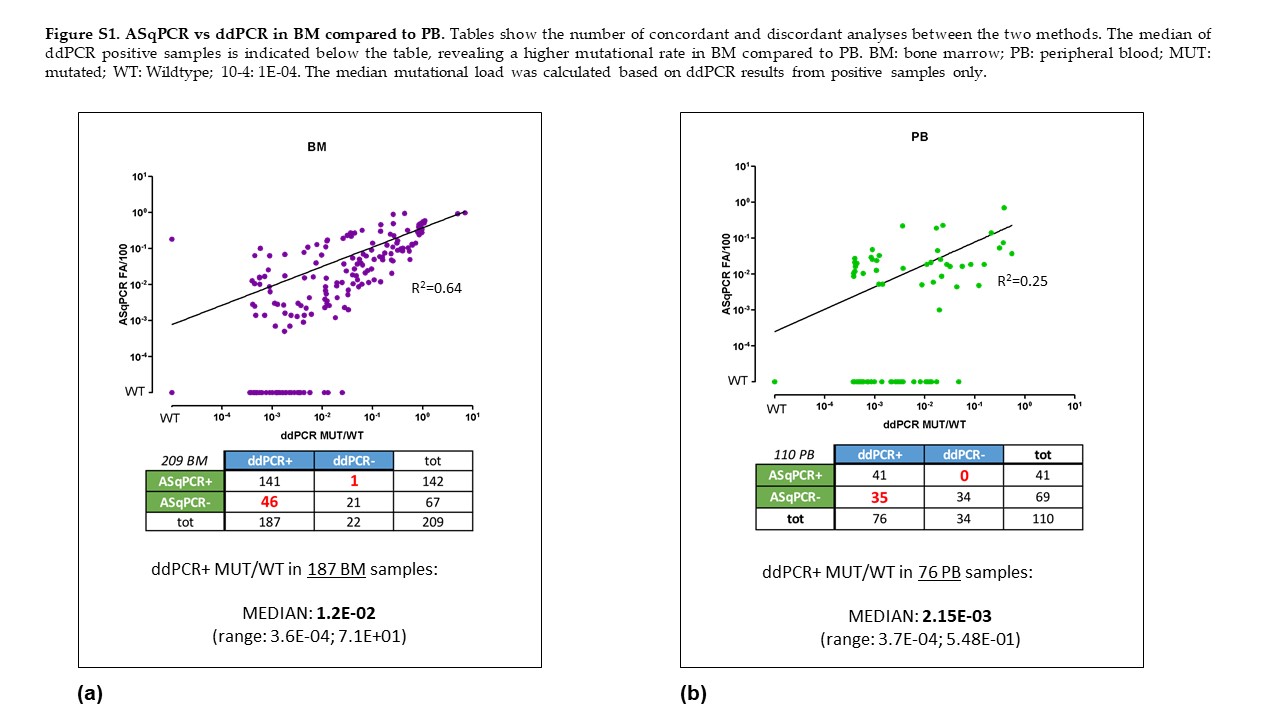

Supplement: Supplementary file 1 [file diagnostics-11-00779-s001.zip › Ferrante M Supplemental proof/FS1.JPG]

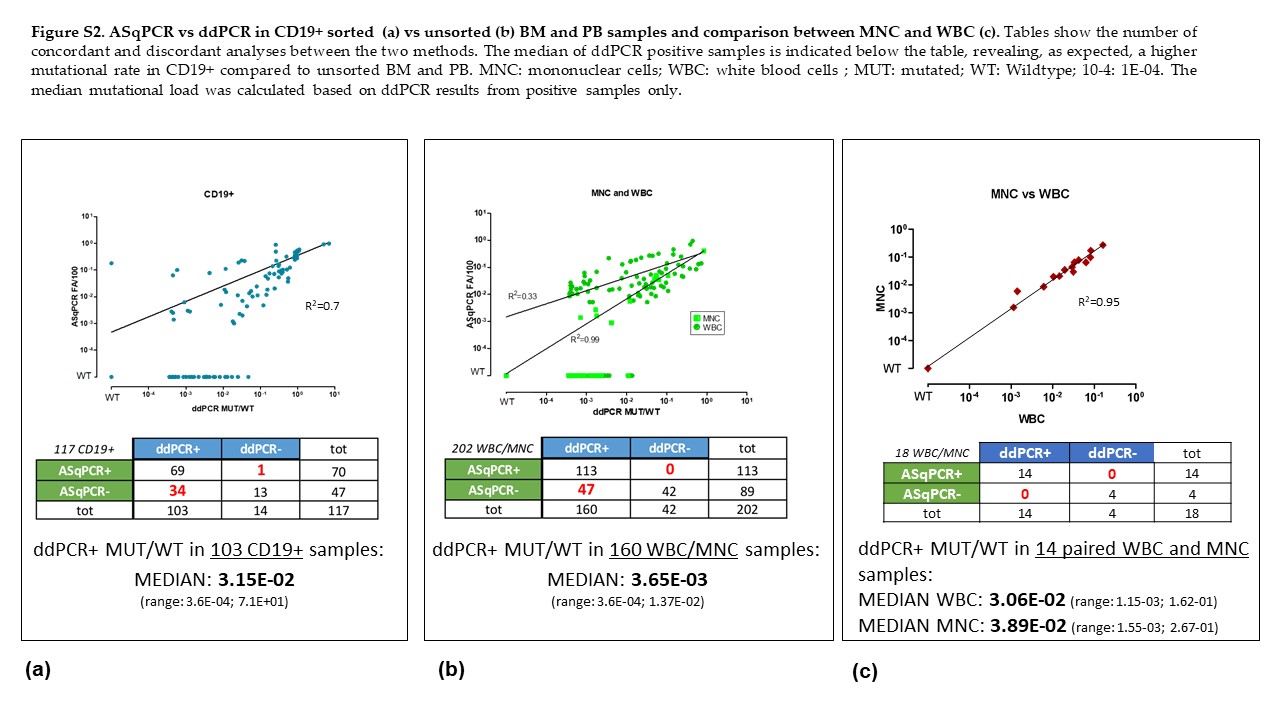

Supplement: Supplementary file 1 [file diagnostics-11-00779-s001.zip › Ferrante M Supplemental proof/FS2.JPG]

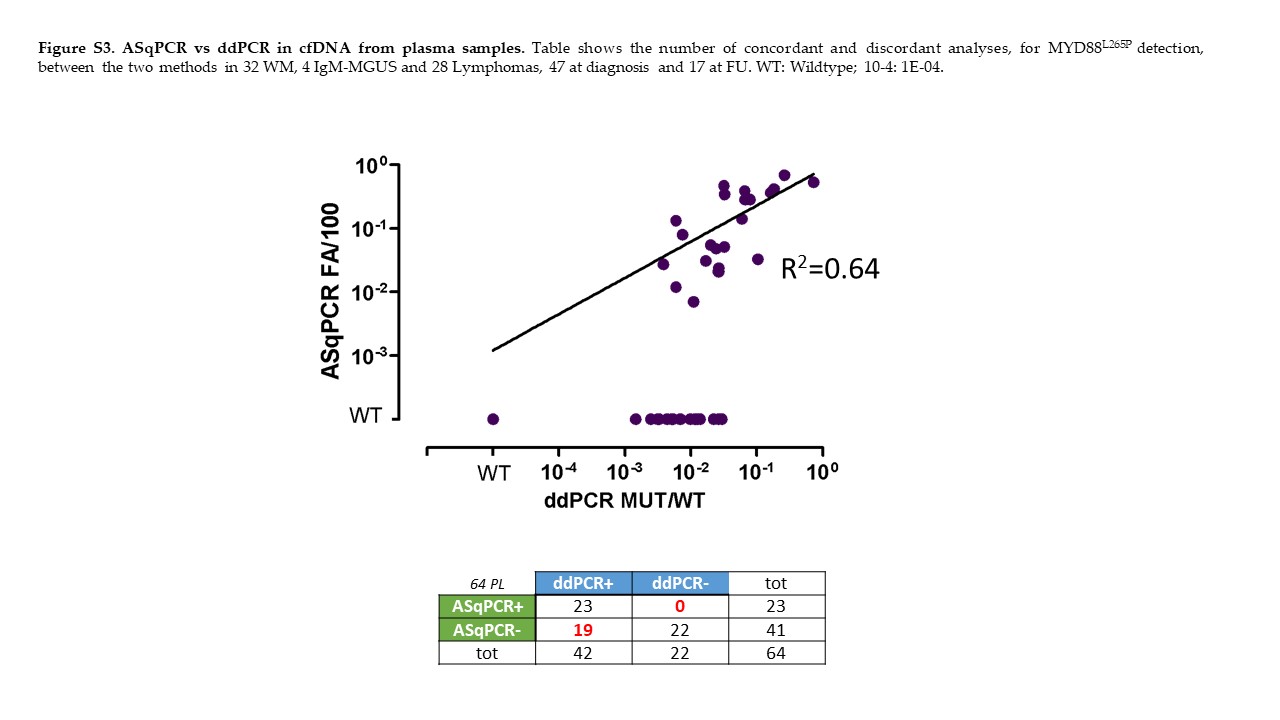

Supplement: Supplementary file 1 [file diagnostics-11-00779-s001.zip › Ferrante M Supplemental proof/FS3.JPG]

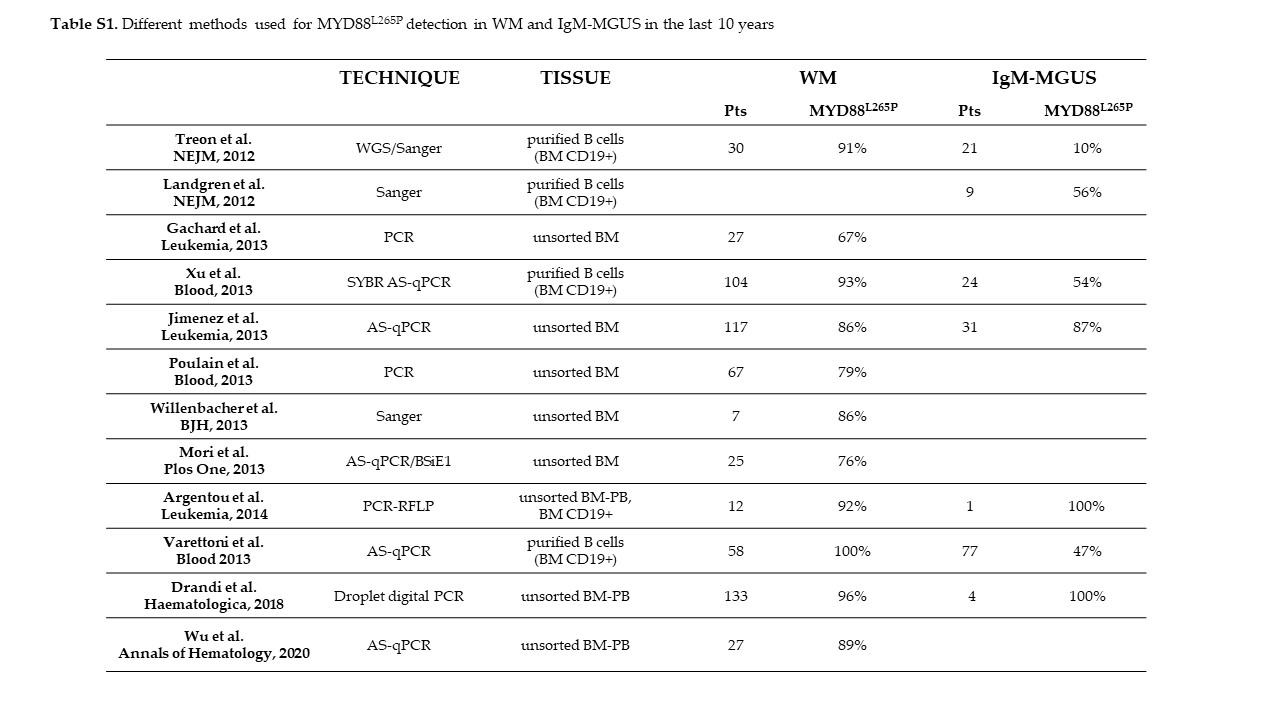

Supplement: Supplementary file 1 [file diagnostics-11-00779-s001.zip › Ferrante M Supplemental proof/TS1.JPG]

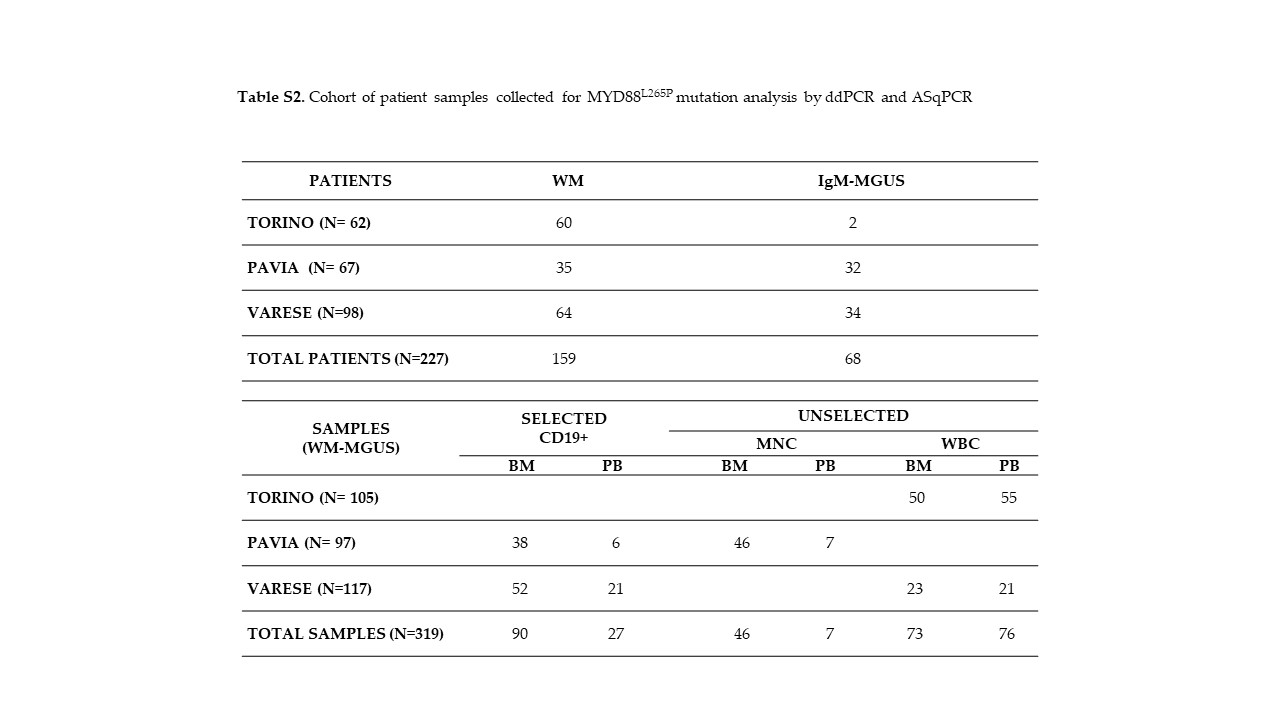

Supplement: Supplementary file 1 [file diagnostics-11-00779-s001.zip › Ferrante M Supplemental proof/TS2.JPG]

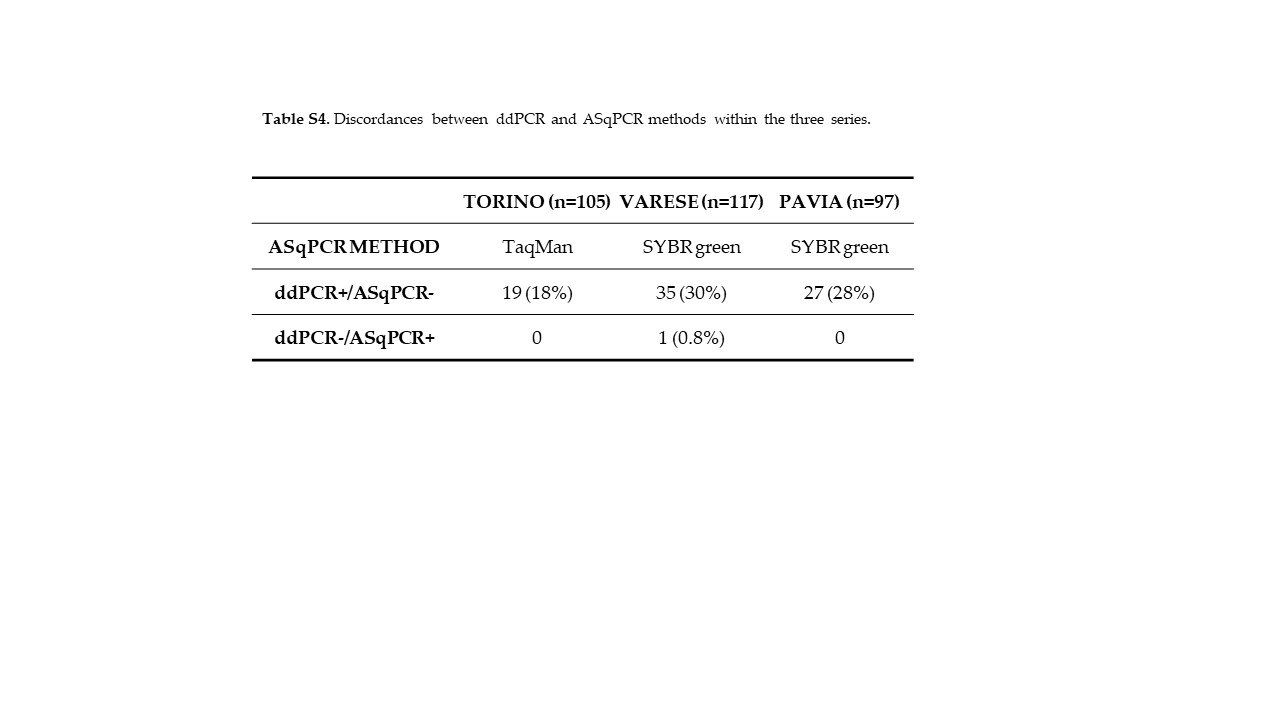

Supplement: Supplementary file 1 [file diagnostics-11-00779-s001.zip › Ferrante M Supplemental proof/TS4.JPG]
